# Chemical Composition of Essential Oil from Four Sympatric Orchids in NW-Italy

**DOI:** 10.3390/plants11060826

**Published:** 2022-03-20

**Authors:** Francesco Saverio Robustelli della Cuna, Pierluigi Cortis, Fabiana Esposito, Antonio De Agostini, Cristina Sottani, Cinzia Sanna

**Affiliations:** 1 Department of Drug Sciences, University of Pavia, Viale Taramelli 12, 27100 Pavia, Italy; fsaveriorobustelli@unipv.it; 2 Casimiro Mondino National Neurological Institute, Via Mondino 2, 27100 Pavia, Italy; 3 Department of Life and Environmental Sciences, University of Cagliari, Via S. Ignazio da Laconi 13, 09123 Cagliari, Italy; antonio.dea@unica.it (A.D.A.); cinziasanna@unica.it (C.S.); 4 Agroecology Lab, Université libre de Bruxelles, 1050 Brussels, Belgium; fabiana.esposito@ulb.be; 5 Environmental Research Center, ICS MAUGERI SPA SB, Institute of Pavia, IRCCS, 27100 Pavia, Italy; cristina.sottani@icsmaugeri.it; 6 Co. S. Me. Se—Consorzio per lo Studio dei Metaboliti Secondari, Via Sant’Ignazio da Laconi 13, 09123 Cagliari, Italy

**Keywords:** *Anacamptis morio*, *Himantoglossum robertianum*, *Ophrys sphegodes*, *Orchis purpurea*, essential oil, alkenes, coumarin, *p*-cresol, pollinators

## Abstract

Orchidaceae is a flowering plant family worldwide distributed known for producing volatile organic compounds (VOCs) which can act as olfactory signals for pollinators. Despite the importance of VOCs in the different reproductive strategies, in the literature there are only a few publications on the characterization of orchids’ volatile profiles. In this study, the essential oils from fresh inflorescences of sympatric orchids *Anacamptis morio*, *Himantoglossum robertianum*, *Ophrys sphegodes* and *Orchis purpurea*, naturally growing in Piedmont (Italy) were isolated by steam distillation and characterized by GC/FID and GC/MS. A number of compounds were identified, with a peculiar distribution in the species: alcohols (range 16.93–50.60%), from which *p*-cresol (range 12.75–38.10%) was the most representative compound; saturated hydrocarbons (range 5.81–59.29%), represented by pentacosane (range 2.22–40.17%) and tricosane (range 0.78–27.48%); long-chain monounsaturated hydrocarbons (range 0.29–5.20%) represented by 9-pentacosene, 11-tricosene, and 1-heneicosene. The structure of positional isomers in linear alkenes was elucidated by derivatization with dimethyl disulfide and MS fragmentation patterns. Coumarin (68.84%) was the dominant compound in *O. purpurea* and was detected in lower concentrations (range 0.21–0.26%) in the other taxa. These volatile compounds may represent a particular feature of these plant species and play an essential role in pollinator interaction.

## 1. Introduction

A large number of plants rely on insects for pollination, by attracting them through visual and tactile cues, as well as by the emission of volatile organic compounds (VOCs) that act as an attractant, both at long- and short-distance [1].

To date, 1700 floral VOCs have been identified in flowers [2,3], mostly terpenoids, phenylpropanoid, benzenoid compounds, and fatty acid derivatives, all contributing to the floral scent of plants. VOCs are capable to enhance sexual reproduction in plants by mediating plant-pollinator interaction [4]. Pollinators easily learn the floral odor of rewarding species by establishing an association between the stimulus and the presence of these rewards [5]. VOCs also play a significant defensive role against herbivores and pathogens, and they preserve plants from oxidative and abiotic stress [6].

Floral volatile profiles are often species-specific, and have been associated with adaption to different pollinators, contributing to the maintenance of reproductive isolation between plant species [7]. Insect pollinators often develop preferences for specific profiles, promoting flower constancy [8]. Unrelated plant species occasionally share floral VOCs in order to attract the same visitors, suggesting a convergent evolution to a unique pollination niche [4].

Orchidaceae is one of the largest families of flowering plant, accounting for around 28,000 species [9]. These plants are known to produce and emit a variety of volatile compounds from different chemical classes [3], which can act as olfactory signals for pollinators depending on their particular reproductive strategy. Nearly one-third of orchid species rely on deceptive strategies to attract pollinators [10,11]. Deceptive species are known to produce complex mixtures of VOCs consisting of more than 100 chemical compounds [12,13]. Out of them, sexually deceptive species, in which pollinator attraction is controlled by olfactory signals, represent a highly specialized example of the role of floral VOCs in plant speciation [14,15]. In the *Ophrys* genus, the sexual deception consists of an sophisticate mimicry that involves, in addition to visual and tactile cues, the production of some VOCs that mimic the female pollinator’s sex pheromone [14,16,17]. The relationship between plant and insect is very often species-specific, thus many *Ophrys* species are pollinated by one or a few insect species. This specific attraction is usually mediated by the pattern of relative amounts of alkenes with different double-bond positions in the floral scent orchids, indicating the importance of unsaturated hydrocarbons for pollinator attraction [7]. However, in some cases, distinct *Ophrys* species growing in sympatry, can attract the same pollinator by producing the same scent bouquet, as documented for example for *O. iricolor* and *O. incubacea* [18]. Contrarily, *O. speculum* attracts its pollinator, the wasp *Campsoscolia ciliata*, by unconventional chemicals, namely hydroxy and oxo acids [19].

Another group of deceptive orchids, namely food-deceptive (including the genera *Anacamptis*, *Orchis* and *Dactylorhiza*) mimic floral traits characteristic of food-providing species [20]. Although the main strategies adopted by these genera rely on visual cues, numerous VOCs have been found in the floral scent of several food-deceptive orchids, which are known to influence the behavior of pollinating insect [21]. As previously observed, in these species, the role of olfactory signals in attracting pollinators may be related to the amount of volatiles emitted [1,22]. In contrast to the sexual-deceptives, food-deceptive orchids have a lower pollinator specialization [23,24], and since pre-zygotic barriers are very weak, they may lead to hybridization when they occur in sympatry [25,26].

Finally, an unusual pollination strategy, called shelter deception, has been employed by the genus *Serapias*. The flowers of these species are nectarless and form a small tube that serves as a shelter for pollinators. Some studies on this genus reported the key role of aliphatic compounds as pre-pollination barriers. These compounds are similar to those used in sexual mimicry in *Ophrys* genus and they are probably used as olfactory signals in *Serapias* genus [27].

Despite the importance of floral scent in the different reproductive strategies, in the literature there are only a few publications on the characterization of orchids’ volatile profiles.

**Figure 1:**
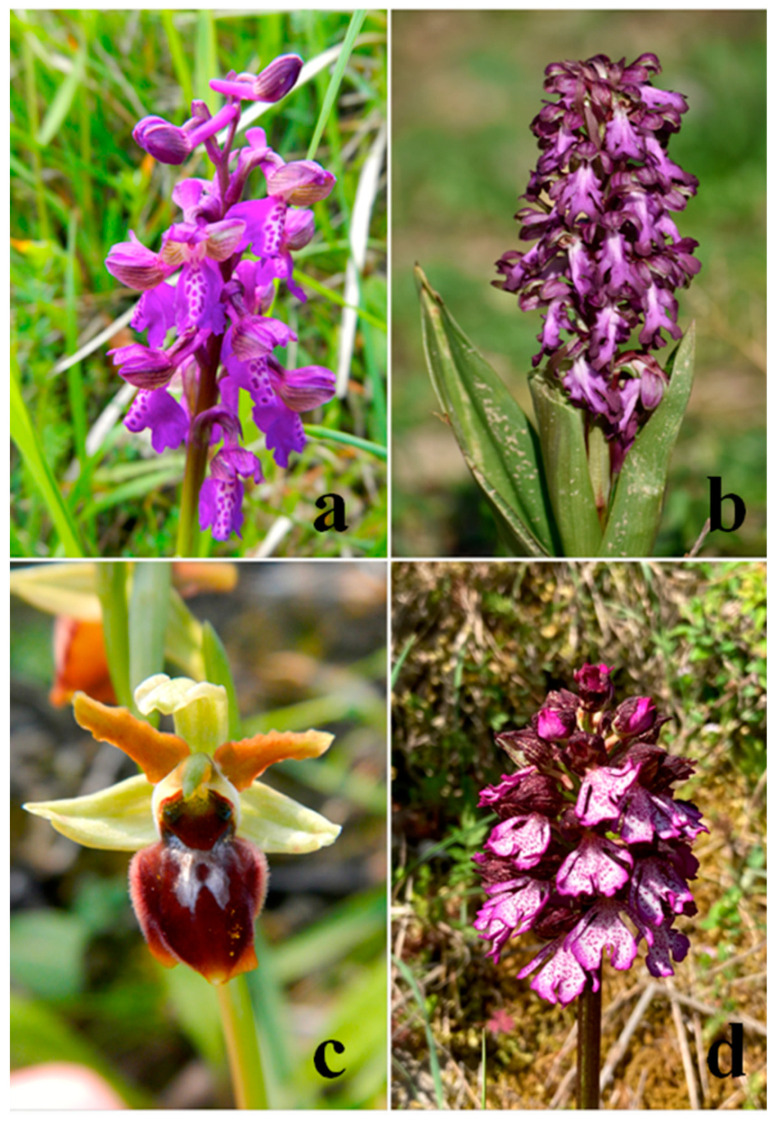
Orchid’s species selected for this study: *Anacamptis morio* (**a**); *Himantoglossum robertianum* (**b**); *Ophrys sphegodes* (**c**) and *Orchis purpurea* (**d**).

Our study aimed at investigating the essential oil profiles of *Anacamptis morio* (L.) R. M. Bateman, Pridgeon and M. W. Chase, *Himantoglossum robertianum* (Loisel.) P. Delforge, *Ophrys sphegodes* (Mill.) and *Orchis purpurea* (Huds.) (Figure 1), naturally growing in Pianlago Ponzone, Piedmont (Italy) to improve the knowledge on the floral scent of these taxa.

## 2. Results and Discussion

The essential oils obtained by steam distillation from fresh inflorescences amounted to 27.1 mg for *A. morio*, 24.9 mg for *H. robertianum*, 19.0 mg for *O. sphegodes* and 15.60 mg for *O. purpurea*. The yields, expressed as weight/dry weight basis, were 0.18%, 0.17%, 0.05% and 0.02%, respectively. Table 1 lists the components identified in the essential oils, reported as percentages of the total essential oil, together with their retention indices (RI) on the Elite-5 MS column, compared to the corresponding values from the literature [28]. Quantitative data were obtained from GC/FID analyses using an internal standard method and assuming an equal response factor for all detected compounds. Information on the identification methods was also provided.

Differences in the qualitative and quantitative composition of the volatile essential oils obtained from the four sympatric Italian orchids can be observed. The essential oils are complex mixtures of volatile compounds whose production can be influenced by several factors such as physiological and seasonal variations, environmental conditions, and genetic factors [30]. In our study we can hypothesize that genetic factors have a greater influence compared to the environmental factors, since the studied species belong to four different genera. Moreover, we have collected all samples in the same area and in the same period of the year, making negligible the environmental and seasonal influence on the observed chemical profiles.

**Figure 2:**
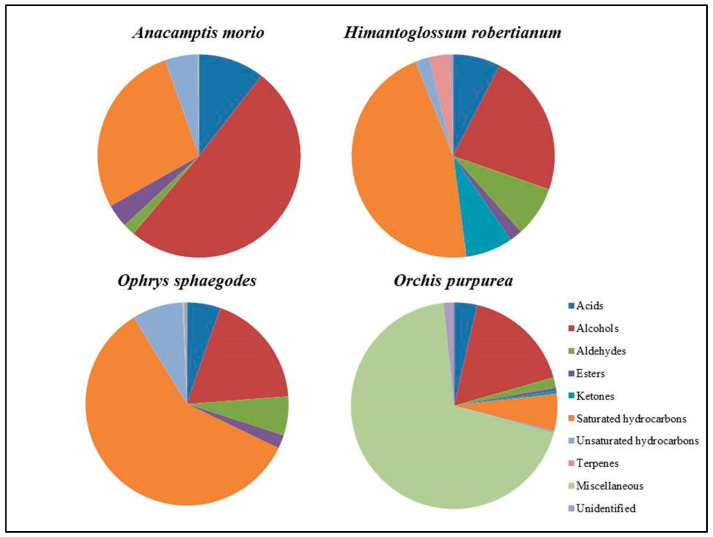
Pie chart of distribution of the classes.

A total of 82 compounds were detected: out of them, 30, 26, 45, and 48 were identified in *A. morio*, *H. robertianum*, *O. sphegodes* and *O. purpurea*, respectively. As shown by the Pie chart (Figure 2), the main constituents belong to saturated hydrocarbons, acids, esters, coumarins, and alcohol classes, with significant differences among the four species.

To be noticed the chemical variability among the food-deceptive species we studied (*A. morio*, *H. robertianum* and *O. purpurea*), confirming the secondary role of chemical cues in food deception strategy if compared to visual and tactile ones. Moreover, the high chemical variability of volatile profiles in non-rewarding species may play a role in bypassing insect learning [1], increasing the reproductive success.

Unexpectedly, except for the high content of unsaturated hydrocarbons, the only sex-deceptive species we studied (*O. sphegodes*) is not characterized by a peculiar volatile profile. Nevertheless, in line with our observations, unsaturated hydrocarbons are considered to play a key role in sex deception of pollinators [14], confirming the relation between chemical composition and sex-deceptive strategy in orchids.

It is worth noting that, there are significant differences between our results and those reported in the literature. In particular, we found a higher amount of saturated hydrocarbons, alcohols and esters, compounds abundant in cuticular waxes in the surface of labella, having the primary function to protect plants from dehydration, but without being involved in the composition of the scent. Probably, these differences are most likely due to different procedures employed in the extraction of the orchid’s scent. In contrast with the solid-phase microextraction (SPME) performed in most literature, we analyzed the chemical profile of the essential oil extracted from the inflorescences by steam distillation.

The essential oils collected from each species are described qualitatively and quantitatively below.

*Anacamptis morio*: the major constituents of the essential oil were found to be alcohols (50.60%), from which *p*-cresol (38.10%), diacetone alcohol (9.04%), and 2,4-di-tert-butyl-phenol (1.39%) were the most abundant compounds. Saturated hydrocarbons (27.70%) represented the second largest class, dominated by pentacosane, tricosane, and tetracosane (17.14%, 7.07%, and 1.22%, respectively). Organic acids, accounting to 10.57%, were represented by hexadecanoic acid (7.54%), followed by heptanoic acid (2.13%), and nonanoic acid (0.52%). A series of unsaturated linear chain hydrocarbons (5.20%) was also identified, of which 1-hexadecene (1.17%) 1-heptadecene (1.13%) and 1-heinecosene (1.11%) were the most abundant compounds. Isopropyl myristate (3.73%) was the only compound belonging to the esters class. Aldehydes (1.94% of the total essential oil) consisted mainly of heptanal (1.33%), nonanal (0.19%), and decanal (0,18%). The essential oil of *A. morio* here reported is strongly different from those obtained by other taxa of this genus. The volatile profiles of *A. coriophora* subsp. fragrans and A. pyramidalis were characterized by a higher percentage of saturated hydrocarbons (81,57% and 52,43%, respectively) and a lower amount of alcohols [31]. In our study we found that the essential oil of *A. morio* lacked terpenes, in contrast to what was reported by Salzmann et al. [32]. These differences are probably due to the different extraction methods. They analyzed the flower scents of *A. morio* by headspace sorption, a rapid and solvent-free method that reduces stress and mechanical damage to the plants, preserving volatile profiles [24]. Salzmann et al. [32] reported monoterpenes as the dominant chemicals, amounting to 50% of the total scent profile in the Italian population, and ranging from 16 to 22% in Swiss samples. Interestingly, the same authors documented low amounts of floral volatiles in *A. morio*, a trait which can be selectively advantageous in food-deceptive species. A strong fragrance may be associated with a non-rewarding flower type, enhancing the discrimination ability of insects and reducing the reproductive fitness of the species [33,34].

*Himantoglossum robertianum*: this species until a few years ago was included in the monospecific genus Barlia and now transferred to the new clade Himantoglossum. As though *Anacamptis morio*, it is a non-rewarding food-deceptive plant that does not contain nectar in its short spur. We found that the main bulk of constituents was represented by saturated hydrocarbons, accounting for 45.97% of the total essential oil, from which pentacosane (40.17%), and tricosane (4.30%) were the most abundant compounds. The second-largest class was characterized by alcohols accounting for 22.68%, from which *p*-cresol (15.28%), diacetone alcohol (4.04%), and 2,4-di-tert-butyl-phenol (1,44%) were the most representative compounds. Aldehydes (8.07%) were represented by nonanal (4.41%), followed by octadecanal (2.80%), and 2,4-decadienal (*E*,*E*) (0.62%). Organic acids, accounting for 7.61%, were represented by hexadecanoic acid (4.94%), followed by heptanoic acid (1.40%), and nonanoic acid (1.27%). α-isoforone (4.21%) and β-forone (3.33%) were the most abundant compounds of the ketone class (7.54%). Terpenes (3.29% of the essential oil) were mainly represented by trans-verbenol (2.40%), terpinen-4-ol (0.61%) and α-terpineol (0.28%). Linear-chain monounsaturated hydrocarbons accounted for 2.28% and consisted of 1-heptadecene (1.38%) and 1-hexadecene (0.90%). To the best of our knowledge there is no previous study on the essential oil of *H. robertianum*. Romano et al. [35] recently analyzed the volatile compounds contained in the floral scent, but they used a different method (HS-SPME), thus their results are strongly different from our ones. They observed a high scent variability in composition and abundance of VOCs both between populations and individuals, but no correlation with geographic distance and environmental variables has been highlighted. Furthermore, compounds such as verbenone identified in Italian populations of *H. robertianum* [35], were not found in the study performed on Spanish populations [36]. The extreme variability in floral emissions of this non-rewarding species can represent an effective strategy to avoid insects learning to associate the floral odor with the lack of nectar. This suggests that there is no adaptation of floral scent to specific pollinators communities [35], as confirmed by the observation of different pollinators belonging to Apoidea (Hymenoptera) and Cetoniidae (Coleoptera) groups for this species.

*Ophrys sphegodes*: saturated hydrocarbons were the main constituents of this essential oil, accounting for 59.29%, from which tricosane (27.76%), pentacosane (12.33%), heneicosane (9.24%), tetracosane (3.33%), and docosane (2.17%) were the most representative compounds. The second-largest class was represented by alcohols accounting for 18.49%, mainly *p*-cresol (12.75%), diacetone alcohol (3.88%), and 2,4-di-tert-butyl-phenol (0.69%). Linear-chain monounsaturated hydrocarbons accounted for 8.12% of the total essential oil, consisting of 9-pentacosene (3.03%), 11-tricosene (1.59%), and 9-tricosene (0.72%). Aldehydes (6.24%) were represented by octadecanal (2.53%), nonanal (1.10%), and heptanal (0.80%). Organic acids represented 5.39% of the essential oil, mainly consisting of nonanoic acid (3.09%), hexadecanoic acid (1.88%), and dodecanoic acid (0.36%). Isopropyl myristate (2.15%) was the only compound belonging to the esters class. This species, commonly known as the early spider orchid, is a sexually deceptive species that attracts specific pollinators by chemical mimicry, by producing the same volatile compounds that constitute sex pheromones of the virgin female of pollinators. In particular, some hydrocarbons and terpenes act as semiochemicals, by triggering mating behavior in pollinators [14,17]. Most of them, especially alkanes and alkenes, were detected both in flowers and in the surface of the female’s cuticle [17], proving their pivotal role in pollinator attraction. These compounds were also found to be biologically active in male olfactory receptors based on gas chromatography with electroantennographic detection (GC-EAD) [14].

*Orchis purpurea*: the essential oil was characterized by a very high content of coumarin (68.84%), a compound having a sweet smell that resembles vanilla. Its derivative, 3,4-dihydrocoumarin, has been also detected but in lower amounts (0.05%). The second-largest class (16.93%) was represented by alcohols, particularly *p*-cresol (12.99%), *p*-vinyl-phenol (2.37%), and 4-methoxy-vinyl-phenol (0.40%). Saturated hydrocarbons (5.81%) were dominated by pentacosane, heptacosane, and heneicosane (2.2%, 1.06% and 0.87%, respectively). Organic acids accounting for 3.63% were represented by hexadecanoic acid (2.14%), followed by tetradecanoic acid (0.59%), and nonanoic acid (0.54%). Aldehydes represented 1.62% of the total essential oil, of which E-15-heptadecenal (0.67%) and nonanal (0.61%) were the most abundant compounds. Ethyl linolenate and ethyl hexadecanoate (0.33%, 0.17%, respectively) were the characterizing compounds of the ester class (0.50%). Linear-chain monounsaturated hydrocarbons accounted for 0.29%, and consisted of 1-hexadecene (0.18%), and 9-pentacosene (0.12%). *O. purpurea* is a food-deceptive non-rewarding species pollinated by generalist pollinators. To the best of our knowledge, there is no previous report on its volatile profile. It is worthy of note the very high percentage of coumarin. Some authors hypothesized a phytoalexin-like defensive role for coumarin [37], although its role in plant-pollinator interactions has not yet been investigated.

**Figure 3:**
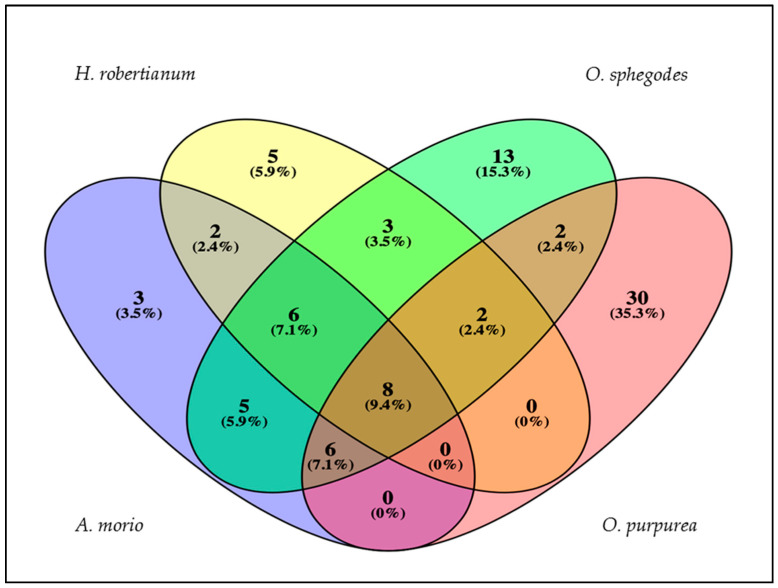
Venn’s diagram shows both the number of compounds shared and unshared/peculiar among the four orchid species. Percentages are referred to the total number of compounds found, not to the relative abundance.

As shown in Figure 3, Venn’s diagram highlighted that 8 compounds were shared among all four sympatric orchids. Differently, some compounds were found to be species-specific. Specifically, three compounds, namely 2-methoxy *p*-cresol, 1-tetradecene, and 1-heneicosene were found only in *A. morio*. Five compounds (β-phorone, α-isophorone, trans-verbenol, exadecen-4-ol, and α-terpineol) were peculiar of *H. robertianum* essential oil. The essential oil of *O. sphegodes* was characterized by 13 peculiar compounds (8.08% of the total essential oil), of which docosane, 11-tricosene, and 9-tricosene were the most abundant ones. *O. purpurea* showed thirty unshared compounds (i.e., heptacosane, hexadecenoic acid, and nonanoic acid) out of a total of 48 components identified.

## 3. Materials and Methods

### 3.1. Plant Material

Inflorescences of the four orchid species were collected in April 2021 in Pianlago Ponzone (Piedmont, Italy, 44°35′21″ N 8°27′37″ E) according to the regional law and with the legal permission of Regional Authorities. Plants were identified according to Chase et al. [38]. A voucher specimen for each species was deposited in the living collection of the Department of Drug Sciences (Pavia, Italy) with the accession numbers ANmo01, HYro01, OPsp01, and ORpu01 for *A. morio*, *H. robertianum*, *O. sphegodes*, and *O. purpurea*, respectively. The flowers were cut and immediately placed in a PVC bag and stored at −20 °C.

### 3.2. Isolation of Essential Oil

Fresh flowers of *A. morio* (14.22 g), *H. robertianum* (14.48 g), *O. sphegodes* (34.65 g) and *O. purpurea* (60.62 g), to which octyl octanoate (98%, Sigma-Aldrich, Inc., St. Louis, MO, USA) was added as internal standard, were steam distilled for 3 h. Steam distillation was performed in the steam distillation system, followed by a solvent extraction, that was necessary to isolate the essential oil from the aqueous phase [31,39]. Briefly, the distillate was extracted three times with 100 mL of methylene chloride, dried over anhydrous Na_2_SO_4_ concentrated with a rotary evaporator, and finally using a gentle stream of N_2_, stored at −20 °C until analysis.

### 3.3. Fractionation and Alkylthiolation of Alkenes

After analyses of the whole essential oil, a portion from each sample was subjected to a selective purification process [37] and alkylthiolation reaction [40]. Briefly, each sample was placed onto a glass column (7 × 30 mm) of silica gel 60, 230–400 mesh (Merck, Darmstadt, Germany), preconditioned with pentane. The non-polar fraction containing hydrocarbons was eluted with 5 mL of pentane and evaporated to dryness with a gentle stream of N_2_. The residue was dissolved in hexane (200 μL), treated with 200 μL of dimethyl disulfide (DMDS) (98%, Sigma-Aldrich, Inc., St. Louis, MO, USA), and 100 μL of iodine (60 mg/mL in diethyl ether). The reaction mixture was held for 4 h at 40 °C, diluted with hexane (1 mL), and washed three times with 5% anhydrous sodium thiosulfate (2 mL). The organic phase was evaporated to dryness, dissolved in hexane, and analyzed by GC/MS. The dimethyl disulfide adducts were identified, and the positions of the methyl sulfide substituents were deduced from the fragmentation pattern.

### 3.4. GC-FID and GC-Ms Analysis

The analyses of essential oils were carried out according to Robustelli della Cuna et al. [31]. DMDS derivatives were analyzed by GC/MS using the same chromatographic equipment with the following operative program: samples (1.0 μL) were injected in “split” mode (30:1) with a column temperature program of 70 °C for 5 min, then increased to 320 °C at 7 °C/min and finally held at this last temperature for 10 min. The relative amount of each component was calculated based on the corresponding GC/FID peak area without response factor correction [31,37,39].

### 3.5. Identification of the Components of the Volatile Fractions

The identification of the volatile oil components was performed by their retention indices (RI) and their mass spectra [28] and by comparison with a NIST database mass spectral library, as well as with literature data [29,41]. Retention indices were calculated by Elite-5MS capillary columns using an n-alkane series (C6–C35) (Sigma-Aldrich, Inc., St. Louis, MO, USA) under the same GC conditions as for the samples.

## 4. Conclusions

In this study, we analyzed the essential oil obtained by steam distillation from inflorescences of four Italian sympatric orchids to increase the knowledge of their volatile profiles. Three of these are food-deceptives (*Anacamptis morio*, *Himantoglossum robertianum*, *Orchis purpurea*), while one is sex-deceptive (*Ophrys sphegodes*). The essential oils were characterized by GC–FID and GC–MS, and our results differed significantly from those found in the literature. These differences could be ascribed to different floral scent extraction procedures, usually through solid-phase microextraction (SPME). Saturated hydrocarbons, alcohols, and esters were abundant in all of the essential oils, although terpenes were absent or discovered in small amounts. Nevertheless, differences in the qualitative and quantitative composition of the volatile essential oils were found among the four species, which are probably due to genetic factors, since the studied species belong to four different genera. A very high variability among food deceptive species has been observed, confirming the secondary role of floral emissions in the attraction of pollinators. In contrast, in *Ophrys sphegodes* we observed the presence of a series of alkanes and alkenes, compounds that are known to act as semiochemicals in pollinator attraction. Interestingly, *Orchis purpurea* showed a very high content of coumarin, a compound that could have a defensive agent, although its role in plant-pollinator interactions has not yet been investigated.

## Figures and Tables

**Table 1:** List of compounds identified in the essential oils obtained from inflorescences of *A. morio*, *H. robertianum, O. sphegodes* and *O. purpurea*.

Compound ^a^	RI Tab ^b^	RI Mean ^c^	*Anacamptis morio* % ^d^	*Himantoglossum robertianum* %	*Ophrys sphegodes* %	*Orchis purpurea* %	*Identification* ^e^
Octane	800	800	−	−	−	0.08 ± 0.01	STD, RI
Hexanal	801	800	−	−	0.24 ± 0.02	−	NIST, RI
2-Hexanol	809	808	0.66 ± 0.11	−	0319 ± 0.05	−	NIST, RI
Furfural	836	831	−	−	−	0.08 ± 0.03	NIST, RI
Diacetone alchol	841	841	9.04 ± 0.09	4.04 ± 0.10	3.88 ± 0.01	−	NIST, RI
Furfuryl alchol	855	855	−	−	−	0.28 ± 0.04	NIST, RI
1-Hexanol	871	870	−	−	−	0.03 ± 0.02	NIST, RI
Heptanal	901	906	1.33 ± 0.07	−	0.80 ± 0.03	0.02 ± 0.01	NIST, RI
Unidentified	−	907	−	−	−	0.31 ± 0.04	−
Benzaldehyde	961	964	0.23 ± 0.11	−	0.18 ± 0.04	0.07 ± 0.03	NIST, RI
Octanal	1001	1003	−	−	0.22 ± 0.01	−	NIST, RI
2-Ethylhexanol	1031	1031	−	−	−	0.26 ± 0.10	NIST, RI
Phenylacetaldehyde	1042	1042	−	−	−	0.05 ± 0.04	NIST, RI
β-Phorone	1044	1045	−	3.33 ± 0.03	−	−	NIST, RI
Heptanoic acid	1069	1068	2.13 ± 0.11	1.40 ± 0.05	0.07 ± 0.03	−	NIST, RI
*n*-Octanol	1068	1070	−	−	0.47 ± 0.07	−	NIST, RI
*p*-Cresol	1076	1073	38.10 ± 0.12	15.28 ± 0.18	12.75 ± 0.08	12.99 ± 0.24	NIST, RI
Nonanal	1105	1105	0.19 ± 0.02	4.41 ± 0.17	1.10 ± 0.08	0.61 ± 0.14	NIST, RI
α-Isophorone	1121	1128	−	4.21 ± 0.12	−	−	NIST, RI
*trans*-Verbenol	1148	1154	−	2.40 ± 0.43	−	−	NIST, RI
Nonenal	1162	1162	−	−	0.37 ± 0.04	0.04 ± 0.01	NIST, RI
Borneol	1169	1166	−	−	−	0.05 ± 0.03	NIST, RI
Terpinen-4-ol	1177	1174	−	0.61 ± 0.05	−	−	NIST, RI
Unidentified	−	1185	−	−	−	0.27 ± 0.05	−
α-Terpineol	1189	1187	−	0.28 ± 0.12	−	−	NIST, RI
*p*-Cimen-8-ol	1192	1192	0.34 ± 0.03	0.63 ± 0.07	−	−	NIST, RI
*p*-Methyl-guaiacol	1192	1193	−	−	−	0.28 ± 0.08	NIST, RI
2-Methoxy *p*-cresol	1198	1198	0.46 ± 0.11	−	−	−	NIST, RI
Decanal	1207	1207	0.18 ± 0.04	0.25 ± 0.09	0.09 ± 0.04	0.03 ± 0.02	NIST, RI
*p*-Vinyl-phenol	1216	1217	0.54 ± 0.04	0.58 ± 0.35	0.51 ± 0.10	2.37 ± 0.02	NIST, RI
2-Phenoxy ethanol	1226	946	0.07 ± 0.02	0.71 ± 0.04	−	−	NIST, RI
3,5-Dimethoxy-toluene	1264	1267	−	−	−	0.15 ± 0.09	NIST, RI
Nonanoic acid	1271	1261	0.52 ± 0.04	1.27 ± 0.04	3.09 ± 0.02	0.54 ± 0.12	NIST, RI
4-Hydroxy-3-methylacetophenone	1292	1308	−	−	−	0.38 ± 0.03	NIST, RI
2,4-Decadienal (*E*,*Z*)	1302	1309	−	−	0.15 ± 0.04	−	NIST, RI
4-Methoxy-vinyl-phenol	1315	1315	−	−	−	0.40 ± 0.54	NIST, RI
2,4-Decadienal (*E*,*E*)	1319	1321	−	0.62 ± 0.07	0.28 ± 0.10	0.03 ± 0.03	NIST, RI
*p*-Hydroxybenzyl alchol	1357	1356	−	−	−	0.11 ± 0.06	NIST, RI
Decanoic acid	1372	1372	−	−	−	0.04 ± 0.02	NIST, RI
Unidentified	−	1379	−	−	−	0.03 ± 0.03	−
3,4-Hydroxycoumarin	1378	1384	−	−	−	0.05 ± 0.03	NIST, RI
β-Damascenone (*E*)	1385	1386	−	−	−	0.07 ± 0.02	NIST, RI
1-Tetradecene	1393	1393	0.68 ± 0.08	−	−	−	MS, RI
Tetradecane	1400	1400	−	−	−	0.05 ± 0.03	STD, RI
Dodecanal	1409	1411	−	−	0.29 ± 0.05	−	NIST, RI
Coumarin	1458	1454	0.26 ± 0.10	−	0.21 ± 0.03	68.84 ± 0.13	NIST, RI
2,4 Di-tert-butylphenol	1518	1516	1.39 ± 0.10	1.44 ± 0.05	0.69 ± 0.07	−	NIST, RI
Unidentified	−	1560	−	−	−	1,05 ± 0.03	−
Dodecanoic acid	1567	1557	0.38 ± 0.03	−	0.36 ± 0.04	0.32 ± 0.04	NIST, RI
1-Hexadecene	1592	1593	1.17 ± 0.07	0.90 ± 0.06	0.37 ± 0.02	0.18 ± 0.07	MS, RI
Heptadecane	1700	1700	−	0.52 ± 0.03	0.68 ± 0.07	−	STD, RI
1-Heptadecene	1755	1759	1.13 ± 0.10	1.38 ± 0.07	0.54 ± 0.05	−	MS, RI
Tetradecanoic acid	1780	1765	−	−	−	0.59 ± 0.08	NIST, RI
3-Octadecene	1785	1785	−	−	0.14 ± 0.04	−	MS, RI
7-Octadecene	1805	1805	−	−	0.43 ± 0.02	−	MS, RI
Unidentified	−	1821	−	0.49 ± 0.05	0.50 ± 0.09	−	−
Isoprpyl myristate	1827	1826	3.73 ± 0.10	2.00 ± 0.15	2.15 ± 0.04	−	NIST, RI
Ciclohexadecane	1880	1881	−	−	−	0.42 ± 0.04	NIST, RI
Nonadecane	1900	1900		0.97 ± 0.09	0.96 ± 0.06	0.12 ± 0.03	STD, RI
Hexadecanoic acid	1960	1959	7.54 ± 0.09	4.94 ± 0.16	1.88 ± 0.03	2.14 ± 0.04	NIST, RI
1-Eicosene	1994	1994	0.66 ± 0.06	−	0.45 ± 0.03	−	MS, RI
Ethyl hexadecanoate	1995	1995	−	−	−	0.17 ± 0.16	NIST, RI
Eicosane	2000	2000	0.52 ± 0.12	−	0.42 ± 0.04	−	STD, RI
Octadecanal	2021	2025	−	2.80 ± 0.18	2.53 ± 0.28	−	NIST, RI
E-15-heptadecenal	2085	2085	−	−	−	0.67 ± 0.12	NIST, RI
1-Heneicosene	2087	2087	1.11 ± 0.11	−	−	−	MS, RI
Heneicosane	2100	2100	0.70 ± 0.30	−	9.24 ± 0.08	0.87 ± 0.05	NIST, RI
Ethyl linolenate	2159	2135	−	−	−	0.33 ± 0.26	NIST, RI
1-Docosene	2195	2195	0.44 ± 0.08	−	0.16 ± 0.05	−	MS, RI
Docosane	2200	2200	−	−	2.17 ± 0.03	−	STD, RI
11-Tricosene	2261	2261	−	−	1.59 ± 0.04	−	MS, RI
9-Tricosene	2279	2277	−	−	0.72 ± 0.06	−	MS, RI
7-Tricosene	2287	2286	−	−	0.42 ± 0.03	−	MS, RI
Tricosane	2300	2300	7.07 ± 0.07	4.30 ± 0.08	27.76 ± 0.06	0.73 ± 0.07	STD, RI
Tetracosane	2400	2400	1,22 ± 0.05	−	3.33 ± 0.05	−	STD, RI
Docosanal	2432	2431	−	−	0.71 ± 0.08	−	NIST, RI
9-Pentacosene	2474	2476	−	−	3.03 ± 0.05	0.12 ± 0.03	MS, RI
7-Pentacosene	2483	2483	−	−	0.28 ± 0.05	−	MS, RI
1-Docosanol	2493	2493	−	−	−	0.18 ± 0.02	NIST, RI
Pentacosane	2500	2500	17.14 ± 0.05	40.17 ± 0.17	12.33 ± 0.03	2.22 ± 0.21	STD, RI
Hexacosane	2600	2600	1.05 ± 0.05	−	1.39 ± 0.05	0.25 ± 0.01	STD, RI
Heptacosane	2700	2700	−	−	−	1.06 ± 0.03	STD, RI
Acids			10.57	7.61	5.39	3.63	
Alcohols			50.60	22.68	18.49	16.93	
Aldehydes			1.94	8.07	6.24	1.62	
Esters			3.73	2.00	2.15	0.50	
Ketones			−	7.54	−	0.45	
Saturated hydrocarbons			27.70	45.97	59.29	5.81	
Unsaturated hydrocarbons			5.20	2.28	8.12	0.29	
Terpenes			−	3,29	−	−	
Miscellaneous			0.26	−	0.21	69.04	
Unidentified			−	0.49	0.50	1.66	

^a^ Compounds are listed in order of their elution on an Elite-5 column. ^b^ Retention Indices according to Adams [28], unless stated otherwise. ^c^ Retention indices determined on an Elite-5 column using a homologous series of n-hydrocarbons. ^d^ (mean + SD of three replicates). ^e^ Method of identification: STD, pure compound; MS, mass spectrum; NIST, comparison with library [29]; RI, retention indices in agreement with literature values.

## Data Availability

Not applicable.
